# Nectar-Secreting and Nectarless *Epidendrum*: Structure of the Inner Floral Spur

**DOI:** 10.3389/fpls.2018.00840

**Published:** 2018-06-20

**Authors:** Małgorzata Stpiczyńska, Magdalena Kamińska, Kevin L. Davies, Emerson R. Pansarin

**Affiliations:** ^1^Faculty of Biology, Botanic Garden, University of Warsaw, Warsaw, Poland; ^2^Department of Botany, University of Life Sciences in Lublin, Lublin, Poland; ^3^School of Earth and Ocean Sciences, Cardiff University, Cardiff, United Kingdom; ^4^Department of Biology, Faculty of Philosophy, Sciences and Literature of Ribeirão Preto, University of São Paulo, São Paulo, Brazil

**Keywords:** *Epidendrum*, Orchidaceae, nectar, nectary, secretory tissues, floral rewards, cuniculus

## Abstract

*Epidendrum*, the largest genus of Neotropical orchids, contains both nectar-secreting and nectarless species. Here, we compare the fine structure of the inner floral spur, termed the cuniculus, in nectariferous (*E. difforme*, *E. nocturnum,* *E. porpax, E. rigidum, E. vesicatum*) and seemingly nectarless (*E. capricornu*, *E. ciliare, E. criniferum, E. pseudepidendrum*, *E. radicans, E. xanthoianthinum*) species. This is the first time for such a detailed investigation of cuniculus structure to be undertaken for *Epidendrum.* Our aim was to characterize features indicative of secretory activity and to ascertain whether flowers presumed to be nectarless produce alternative pollinator food-rewards. The cuniculus is formed by fusion of the basal part of the labellum and column and extends alongside the ovary and transmitting tract. Our study indicates that all investigated species produce nectar or nectar-like secretion to varying degrees, and no alternative pollinator food-rewards were observed. Even though macroscopic investigation of presumed rewardless species failed to reveal the presence of secretion within the cuniculus, close observations of the cells lining the cuniculus by LM, SEM, and TEM revealed the presence of cuticular blisters and surface material. Moreover, the similarity of both the thick tangential cell walls (with the exception of *E. vesicatum*) and organelle complement of cuniculus epidermal cells in both copiously nectariferous species and those producing only small quantities of surface secretion confirmed the presence of secretory activity in species generally regarded to be rewardless. The secretory character was particularly obvious in the cells of the cuniculus of *E. nocturnum,* but also in *E. ciliare, E. radicans* and *E. xanthoianthinum,* since electron-dense cytoplasm and mitochondria, ER and secretory vesicles were abundant. Furthermore, cell wall protuberances occurred in *E. nocturnum*, which was indicative of intense transmembrane transport. This investigation highlights the need to examine more closely whether *Epidendrum* spp. considered to lack food-rewards based solely on macroscopic examination really are rewardless and deceptive.

## Introduction

Orchids offer their pollinators a variety of floral food-rewards, such as nectar, oil and edible trichomes, with many more producing non-food rewards, such as fragrances, waxes and resins. Based on analyses by Neiland and Wilcock (1998), the presence of nectar in both temperate and tropical orchids can increase their reproductive success (fruit set). In Orchidaceae, nectar is the most common floral food-reward, and here, perigonal nectaries located on the labellum predominate (Bernardello, 2007; Davies and Stpiczyńska, 2008). They may occur in shallow depressions, as in *Epipactis* (Pais, 1987; Kowalkowska et al., 2015), on the labellar callus, as in *Maxillaria* *anceps* (Davies et al., 2005), in the median furrow of the labellum, as in *Listera* (van der Cingel, 2001) and *Bulbophyllum* (Stpiczyńska et al., 2015, 2018), in the labellum base, as in *Cleistes* (Pansarin et al., 2012), *Elleanthus* (Nunes et al., 2013) and *Psilochilus* (Pansarin and Amaral, 2008a), but also on the column, as in *Maxillaria coccinea* and *Ornithidium sophronitis* (Stpiczyńska et al., 2004, 2009), or in the mentum, as in *Dendrobium finisterrae* (Kamińska and Stpiczyńska, 2011). However, the most frequently encountered type of nectary, occurring both in this enormous family and also in other angiosperms, is the nectary spur, which is present in at least 0.60% of angiosperm genera (Mack, 2013; Mack and Davis, 2015). Nectary spurs of various lengths occur as outgrowths of the labellum in representatives of Aeridiinae (Davies and Stpiczyńska, 2008; Stpiczyńska et al., 2011), Maxillariinae (Davies and Stpiczyńska, 2007), Orchidinae (Stpiczyńska, 2003; Bell et al., 2009), and Spiranthinae (e.g., Pansarin and Ferreira, 2015). In *Anacamptis pyramidalis* f. *fumeauxiana* (Orchidinae), in addition to the spur formed at the base of the labellum, two spurs originating from lateral sepals are present (Kowalkowska et al., 2012). In Laeliinae, the nectary, if present, is represented in the majority of cases by a cuniculus – an atypical inner spur formed by fusion of the column and labellum throughout their length, and which runs deep alongside the transmitting tract and ovary.

Regardless of taxonomic position and the presence or absence of floral rewards, the spurs in Orchidaceae studied to date were lined by flat epidermal cells (e.g., *Schoenorchis gemmata* – Stpiczyńska et al., 2011), or conversely, the epidermis was papillose (e.g., *Ascocentrum*) or trichomatous (e.g., *Angraecum germinyanum*, *Papilionanthe vandarum, Platanthera, Dactylorhiza, Brassavola*) (Stpiczyńska, 2003; Davies and Stpiczyńska, 2008; Bell et al., 2009; Stpiczyńska et al., 2010, 2011, respectively). Beneath the secretory epidermis occurred one to several layers of small subepidermal parenchyma cells. Published, detailed, microscopical analyses revealed diverse sculpturing and variable thickness in the cuticle overlying the secretory epidermal cells. Cuticular blisters were observed in *Platanthera* (Stpiczyńska, 2003) and *Schoenorchis gemmata* (Stpiczyńska et al., 2011), but pores were rarely recorded (e.g., *Brassavola* *flagellaris* – Stpiczyńska et al., 2010). Cell walls were predominantly thin or of moderate thickness, with the exception of ornithophilous *Ascocentrum curvifolium* (Stpiczyńska et al., 2011) and moth-pollinated *Brassavola* *flagellaris* (Stpiczyńska et al., 2010). The cells were interconnected by numerous plasmodesmata. Generally, the ultrastructure of secretory cells of the spur conformed with that of typical nectary cells (Nepi, 2007). These cells contained dense cytoplasm with numerous mitochondria, ER profiles, dictyosomes and secretory vesicles (Stpiczyńska, 2003; Davies and Stpiczyńska, 2008; Stpiczyńska et al., 2010, 2011). Additionally, they often contained plastids with prominent starch grains (e.g., *Ascocentrum* – Stpiczyńska et al., 2011), or were completely starchless throughout the lifespan of the flower (e.g., *Gymnadenia* – Stpiczyńska and Matusiewicz, 2001). In *Papilionanthe vandarum,* starchless plastids contained large deposits of phenolic-like material (Stpiczyńska et al., 2011).

*Epidendrum* L. is the largest genus of tribe Epidendreae, subtribe Laeliinae, and according to the APG IV website (Stevens, 2001 onward), comprises 1425 species. It is distributed from the southeastern United States of America to northern Argentina (Hágsater and Soto-Arenas, 2005). It comprises both food-rewarding and food-deceptive species (Pansarin, 2003; Pansarin and Amaral, 2008b; Pansarin and Pansarin, 2014, 2017). Although its flowers are visited by a wide range of pollinators, moths and butterflies are the most frequently recorded, and according to Pinheiro and Cozzolino (2013), this kind of specialization (together with certain novel vegetative characters) may represent key innovations that led to the enormous degree of speciation found in this genus. Ornithophily has been reported for *E. cinnabarinum*, *E. ibaguense,* and *E. pseudepidendrum* (van der Pijl and Dodson, 1969; van der Cingel, 2001). Orange-red or yellow flowers are attributed to butterfly-pollinated species, whereas whitish to pale green, highly fragrant flowers are predominantly moth-pollinated (van der Pijl and Dodson, 1969; van der Cingel, 2001). In some moth-pollinated species, scent is produced by osmophores (Pansarin and Pansarin, 2017). *Epidendrum densiflorum* (= *E. paniculatum*) is pollinated by both butterflies and Arctiidae moths (Pansarin, 2003), whereas *E. avicula*, is pollinated by several species of micro-moths, as well as Tipulidae or crane flies (Pansarin and Pansarin, 2017). In fact, many *Epidendrum* species have a long cuniculus and are thus adapted for pollination by Lepidoptera (Pansarin, 2003; Pansarin and Amaral, 2008b; Fuhro et al., 2010; Pansarin and Pansarin, 2017). Conversely, although other members of Laeliinae have also long been considered to possess a cuniculus (e.g., Dressler, 1993), some taxa, such as *Amblostoma* and *Lanium*, both currently included in *Epidendrum sensu lato*, lack this character (Pansarin and Pansarin, 2014, 2017). Study of the reproductive biology of *E. tridactylum*, a member of the *Amblostoma* group, demonstrated that this species produces fragrant and rewardless flowers, and attracts dipterans that drink the extra-floral nectar produced at the base of the floral bracts (Pansarin and Pansarin, 2014). As in *E. tridactylum*, the flowers of *E. avicula* lack a cuniculus, and the nectary is located at the base of the labellum, inside a tube formed by the labellum and column. As a consequence, insects possessing a short but thin proboscis (i.e., flies and micro-moths) are the most effective pollinators of these orchids (Pansarin and Pansarin, 2017).

In the majority of *Epidendrum* spp., insects searching for nectar insert their proboscides into the cuniculus. Since the entrance to the cuniculus has a keyhole-like structure, such behavior causes the pollinator to become temporarily detained. The traumatized insect thus avoids revisiting the same inflorescence, thereby reducing geitonogamy, or pollen loss in the case of self-incompatible species (Dressler, 1981; Pansarin and Pansarin, 2017). As in many other orchids, flowers of *Epidendrum* are infrequently visited, and low fruit set is common (Adams and Goss, 1976; Ackerman and Montalvo, 1990; Almeida and Figueiredo, 2003; Pansarin and Amaral, 2008b; Fuhro et al., 2010; Pinheiro et al., 2010, 2011).

Despite the presence of a cuniculus, nectar has only rarely been found in *Epidendrum*, and to date, its presence has been recorded only for *E. difforme* (Goss, 1977), *E. compressum*, *E. schlechterianum*, *E. strobiliferum* (Braga, 1977) and *E. avicula* (Pansarin and Pansarin, 2017).

It should be emphasized that reward-producing and rewardless *Epidendrum* species have so far mainly been distinguished by macroscopic observation for the presence or absence of nectar within the inner spur (Almeida and Figueiredo, 2003; Hágsater and Soto-Arenas, 2005; Pansarin and Amaral, 2008b). Detailed structural studies of the cuniculus are scarce, particularly in species where nectar appears to be absent. This is the first time for such a detailed investigation of cuniculus structure to be undertaken for *Epidendrum.* For this study, we selected 11 species of *Epidendrum* that differ in their type of pollination syndrome. The aim of this research is to: (i) compare the structure of the cuniculus in nectariferous species of *Epidendrum* and those regarded to be nectarless; (ii) explore whether the presence of nectar and the structure of the cuniculus are correlated; (iii) check whether flowers assumed to be nectarless produce alternative pollinator rewards.

## Materials and Methods

The majority of plants used in this study were grown at the Botanic Garden of the University of Warsaw, Poland. They include nectar-secreting *Epidendrum difforme* Jacq.*, E. nocturnum* Jacq., *E. porpax* Rchb. f., *E. rigidum* Jacq., and seemingly nectarless *E. capricornu* Kraenzl., *E. ciliare* L., *E. criniferum* Rchb. f., *E. pseudepidendrum* Rchb. f.*, E. radicans* Pav. ex Lindl. and *E. xanthoianthinum* Hágsater. The sole exception was the nectar-secreting *E. vesicatum* Lindl. which was collected in the city of Blumenau, state of Santa Catarina, South Brazil and cultivated at the LBMBP Orchid House, University of São Paulo, Ribeirão Preto, Brazil. The species cultivated at the Botanic Garden of the University of Warsaw were grown in a glasshouse at 25°C, and those which flowered in autumn/winter (*Epidendrum capricornu, E. ciliare E. difforme, E. nocturnum, E. porpax, E. rigidum*) were provided with a photoperiod comprising 12 h light and 12 h darkness. AGRO, PILA, MT WLS400W-Z-00 lamps were used to supplement light during the day. The study was conducted on 1-2 plants of each species, and 5 flowers each were used for microscopical analysis. Abbreviations of authorities for plant names follow Brummitt and Powell (1992) throughout.

The position of the cuniculus and the presence of nectar in longitudinally sectioned flowers on the first day of anthesis were determined by means of a Nikon SMZ100 stereomicroscope. The structure of the tissues surrounding the cuniculus was subsequently examined using light microscopy (LM), including fluorescence microscopy (FM), scanning electron microscopy (SEM) and transmission electron microscopy (TEM). The number of vascular bundles supplying the tissues surrounding the cuniculus was recorded based on transverse sections of the flower taken at the level of insertion of the perianth segments. We considered vascular bundles present in parenchyma surrounding the cuniculus, but not those located near the transmitting tract.

For microscopical observations, pieces of ovary, together with the cuniculus, were excised and fixed in 2.5% (v/v) glutaraldehyde/4% (v/v) formaldehyde in phosphate buffer (pH 7.4; 0.1 M) for 2 h at 4°C, washed three times in phosphate buffer and post-fixed in 1.5% (w/v) osmium tetroxide solution for 1.5 h at 0°C. The fixed material was then dehydrated using a graded ethanol series, and infiltrated and embedded in LR White resin (LR White acrylic resin, medium grade, Sigma). Following polymerization at 60°C, sections were cut at 70 nm for TEM using a Reichert Ultracut-S ultramicrotome and a glass or diamond knife, stained with uranyl acetate and lead citrate (Reynolds, 1963) and examined using a FEI Tecnai Spirit G2 transmission electron microscope, at an accelerating voltage of 90 kV.

Semi-thin sections (0.9–1.0 μm thick) were prepared for LM and FM. For general histology, they were stained with a 1:1 solution of 1% (w/v) aqueous methylene blue: 1% (w/v) aqueous azure II (MB/AII) for 5–7 min.

Histochemical tests were used to detect the presence of lipids and starch in the tissues by treating them with a saturated ethanolic solution of Sudan III and with IKI solution, respectively, followed by examination using a Nikon E-200 or Nikon Eclipse 90i light microscope. The periodic acid-Schiff (PAS) reaction was also employed to detect the presence of insoluble polysaccharides (Jensen, 1962). Semi-thin sections were also treated with auramine O (Gahan, 1984) and examined using FM with FITC filter (excitation light 465–495 nm, barrier filter 515–555 nm) to detect the presence of lipid. A UV2B filter (Nikon) was used to check for chlorophyll autofluorescence. Micrometry and photomicrography were accomplished by means of a Nikon Eclipse 90i (NIS-Elements AR software) or a Stereozoom Leica S8 APO stereomicroscope, in conjunction with a PC employing IM50 image analysis software. For TEM images, the FEI Tecnai Spirit G2 TEM Imaging & Analysis computer program was used. Thicknesses of cell wall and cuticle were measured only for species on which TEM analysis was performed, and the mean calculated (*n* = 10 measurements ± SD).

For SEM, fixed pieces of the flower, cut longitudinally to expose the cuniculus, were dehydrated and subjected to critical-point drying using liquid CO_2_. They were then sputter-coated with gold and examined using a Vega II LS scanning electron microscope at an accelerating voltage of 10 kV.

## Results

### Species With Nectar Visible Upon Macroscopic Observation

**FIGURE 1:**
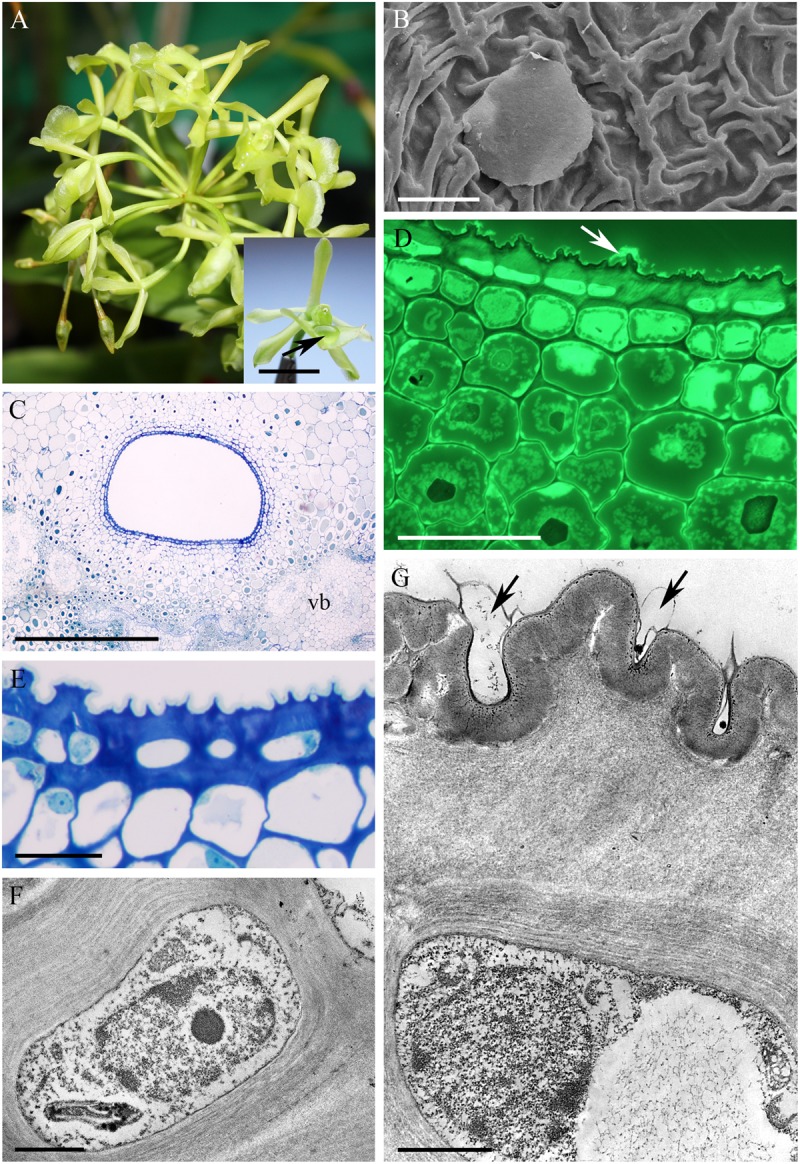
*Epidendrum difforme*. **(A)** Inflorescence. Insert shows flower with droplet of nectar (arrow). **(B)** Convoluted cuticle on surface of epidermal cells with nectar residue. **(C)** Transverse section through cuniculus showing small epidermal cells enclosing the lumen, and parenchyma cells with intravacuolar phenolic-like contents (MB/AII). **(D)** Residues of nectar (arrow) on surface of cuticle. Note thick epidermal cell walls and intravacuolar material (auramine O). **(E)** Detail of epidermis and subepidermal parenchyma (MB/AII). **(F)** Protoplast of epidermal cell. Note the large nucleus and starchless plastid. **(G)** Detail of cell wall and cuticle with associated surface secretion (arrows) of epidermal cells lining the cuniculus. Scale bars: A = 1 cm; B,E = 20 μm; C = 500 μm; D = 50 μm; F,G = 2 μm.

The cuniculus of the light-green flowers of *Epidendrum difforme* was 10 mm long and contained nectar. The entire inner surface of the cuniculus was coated with nectar. A droplet of nectar was also visible on the adaxial surface of the labellum (**Figure 1A**). The flowers did not produce perceptible fragrance. Epidermal cells lining the cuniculus were flat along the whole length of the cuniculus, with coarse cuticular ridges (**Figures 1B–E,G**). Large deposits of secreted material were present on their surface (**Figure 1B**). Transverse sections revealed the thick (7.42 μm ± 1.44), lamellate, cellulosic walls of epidermal cells (**Figures 1E–G**), and the irregular outline of the outer tangential wall. This was due to numerous wall protuberances. The overlying cuticle was 1.02 μm ± 0.12 thick (**Figures 1D,E,G**). Deposits of electron-translucent material were present beneath distensions of the cuticle, and similar material also occurred on the surface of the epidermis (**Figures 1D,G**). The underlying, single-layered, secretory parenchyma had only slightly thickened tangential walls. Protoplasts of epidermal cells were electron dense (**Figure 1F**) and these, in semi-thin sections, stained intensely with MB/AII (**Figure 1E**). Protoplasts of subepidermal parenchyma were also electron dense, but contained relatively large vacuoles. Typical ground parenchyma cells with thin cell walls, a thin layer of parietal cytoplasm, and a large vacuole, occurred ventral to the cuniculus. Plastids in epidermal, subepidermal and ground parenchyma cells only occasionally contained minute starch grains. However, they contained numerous electron-dense globules. Collateral vascular bundles (three main and tree smaller bundles located alternately) embedded in the ground parenchyma did not penetrate the secretory tissue. Parenchyma cells contained intravacuolar deposits of phenolic-like material (**Figures 1C–E**).

**FIGURE 2:**
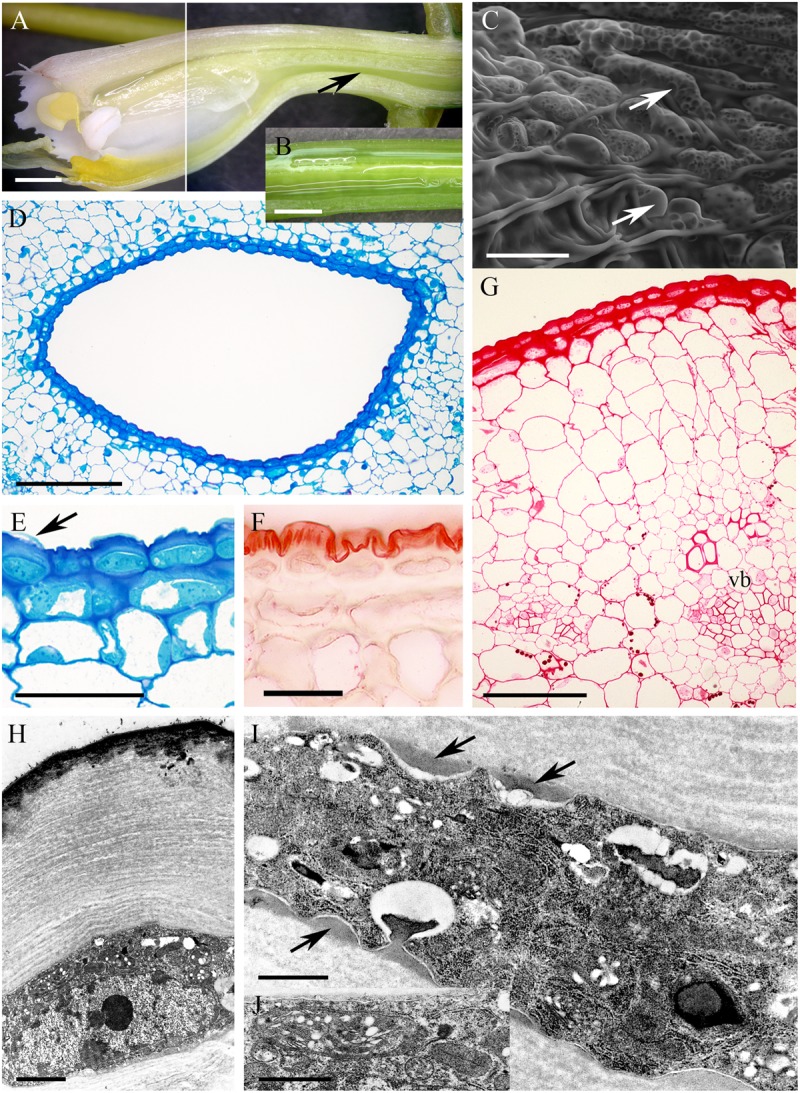
*Epidendrum nocturnum*. **(A)** Longitudinal section of anterior part of the flower showing cuniculus (arrow). **(B)** Detail of cuniculus with copious nectar. **(C)** Epidermal cells with cuticle ridges and cuticular blisters with secretion (arrows). **(D)** Transverse section showing epidermis and subepidermal parenchyma of cuniculus (MB/AII). **(E)** Detail of thick-walled epidermal cells with thin cuticle. Arrow indicates cuticular blister and nectar residues (MB/AII). **(F)** Cuticle lining cuniculus stained with Sudan III. **(G)** PAS reaction stains thick walls of epidermis; large starch grains are located close to vascular bundles. **(H)** Detail of thick outer cell wall and thin cuticle. Note dense protoplast of epidermal cell with large nucleus and plastids. **(I)** Protuberances (arrows) of thick cell wall of epidermal cell. The electron-dense cytoplasm contains numerous ER profiles and plastids. **(J)** Detail of cytoplasm of epidermal cell with plastid containing minute starch grains. A,B = 2 mm; C, E,F = 50 μm; D = 200 μm; G = 100 μm; H = 2 μm; I,J = 1 μm.

In *Epidendrum nocturnum*, the flowers are greenish-white and fragrant. The cuniculus was 46 mm long and contained copious nectar (**Figures 2A,B**). The epidermis enclosing the cuniculus was composed of small, slightly convex cells (**Figures 2C–G**). The hypodermal cells were also small, and beneath these occurred typical ground parenchyma supplied by three main collateral vascular bundles and several phloem strands (**Figure 2G**). Epidermal and hypodermal cells possessed thick (9.91 μm ± 7.13), cellulosic, lamellate, tangential walls (**Figures 2E–I**). Numerous protuberances projected from the cell walls (**Figure 2I**). The cuticle overlying the epidermis was relatively thin (0.60 μm ± 0.21), as seen in transverse section (**Figure 2F**), and bilayered, the outer layer being lamellate and electron dense (**Figure 2H**). Coarse cuticular ridges and distensions were visible using SEM, and secretory residues were present on the surface of the cuticle (**Figure 2C**). Epidermal and subepidermal parenchyma cells contained dense cytoplasm and large nuclei (**Figures 2E,H–J**). Dictyosomes, mitochondria, ER profiles and numerous secretory vesicles were present in the cytoplasm (**Figures 2I,J**). The plasmalemma was invaginated, and the periplasmic space contained secretory material (**Figure 2I**) or secretory vesicles. In epidermal cells, plastids contained only minute starch grains (**Figures 2I,J**) that were not detectable with the PAS reaction, but starch was more abundant in the ground parenchyma adjacent to vascular bundles (**Figure 2G**). Chloroplasts occurred exclusively in ground parenchyma cells.

**FIGURE 3:**
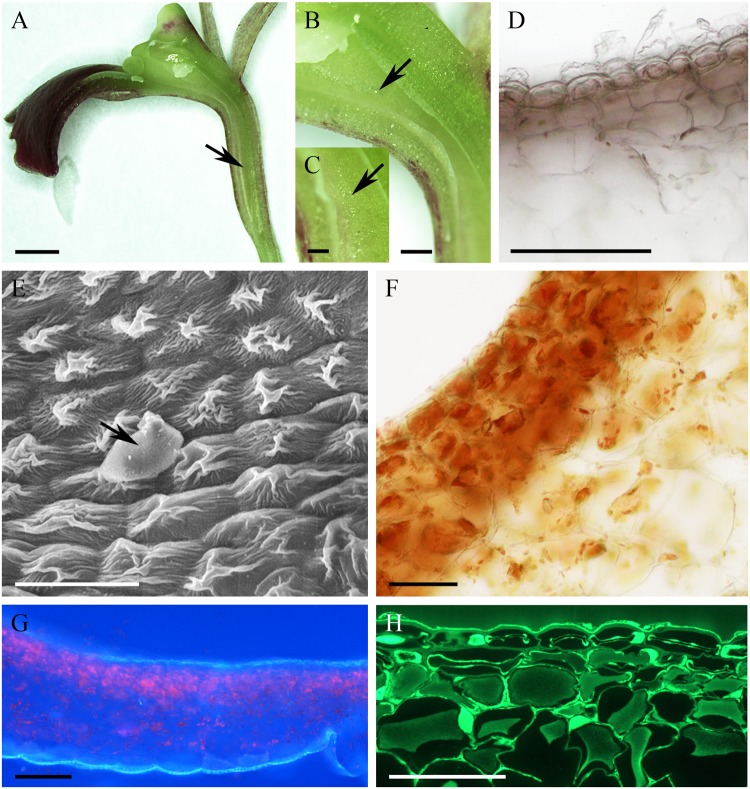
*Epidendrum porpax*. **(A)** Longitudinal section of flower. Cuniculus indicated by arrow. **(B,C)** Details of cuniculus with small droplets of nectar (arrows). **(D)** Epidermal cells with surface secretion and subepidermal parenchyma (unstained, hand-cut section,). **(E)** Surface of epidermis with nectar residue (arrow). **(F)** Section stained with IKI; note starchless plastids. **(G)** Longitudinal section of cuniculus, the lumen visible at its top. Autofluorescence of chlorophyll located in subepidermal and ground parenchyma on exposure to UV. **(H)** Thin cuticle of epidermal cells with secretory residues. Beneath the epidermis occur large, thin walled parenchyma cells (auramine O). Scale bars: A = 2 mm; B = 500 μm; C = 200 μm; D,G = 100 μm; E,F,H = 50 μm.

The cuniculus of the small, non-fragrant, brown-green flowers of *Epidendrum porpax* was 6 mm long. It had a relatively wide entrance, but tapered toward its base (**Figures 3A,B**). Minute droplets of nectar were visible on the inner surface of the cuniculus using a stereomicroscope, and nectar residues were visible on the cuticle surface using SEM and LM (**Figures 3C–F**). Epidermal cells lining the cuniculus were smaller than those of the hypodermis, and only the outer tangential walls of the epidermal cells were thickened (**Figures 3D,H**). The cuticle overlying the epidermis was thin, ridged, and occasionally distended (**Figure 3E**). Starch was absent from the epidermis and subepidermal parenchyma, but present in ground parenchyma cells (**Figure 3F**), whereas chloroplasts occurred in the subepidermal parenchyma cells (**Figure 3G**). Both epidermal and parenchyma cells contained intravacuolar phenolic-like compounds (**Figure 3H**). Three collateral vascular bundles ran through the ground parenchyma.

**FIGURE 4:**
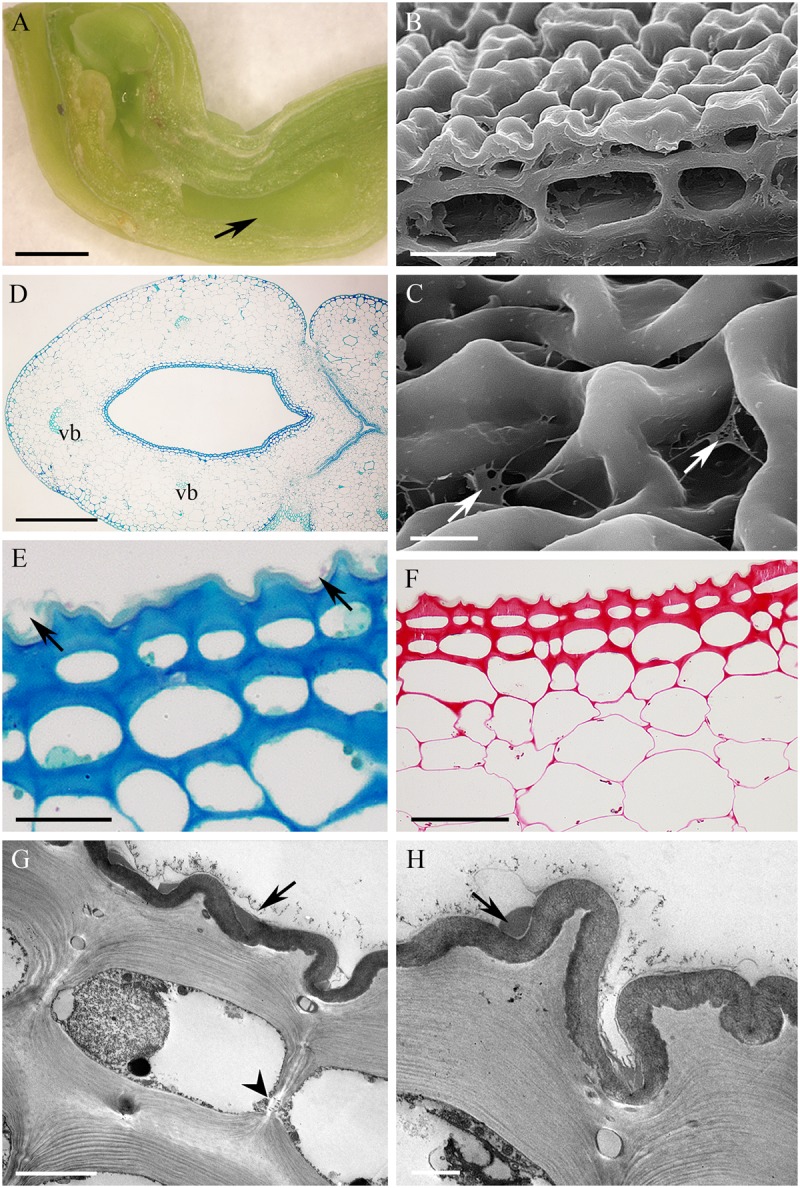
*Epidendrum rigidum*. **(A)** Longitudinal section of flower showing cuniculus (arrow). **(B)** Detail of small, thick-walled epidermal cells with convoluted cuticle, and larger subepidermal parenchyma cells. **(C)** Detail of cuticle with nectar residues (arrows). **(D)** Transverse section of ovary showing cuniculus enclosed by epidermis and parenchyma containing vascular bundles (MB/AII). **(E)** Detail of collenchymatous epidermis and subepidermal parenchyma. Note thin cuticle with secretory residues (arrows). **(F)** The PAS reaction stains cell walls and occasional starch grains in ground parenchyma. **(G)** Epidermal cell lining cuniculus, with large nucleus and parietal cytoplasm containing osmiophilic globules. Secreted surface material occurs on the cuticle (arrow). Plasmodesmata in anticlinal cell wall marked with arrowhead. **(H)** Detail of outer wall of epidermal cell lining cuniculus, showing cuticle with nectar residues (arrow) and cavity in middle lamella. Scale bars: A = 1mm; B,C,E = 20 μm; D,G = 5 μm; D = 500 μm; F = 50 μm; H = 2 μm.

In the small, non-fragrant, green flowers of *Epidendrum rigidum*, the cuniculus was 8 mm long, with a narrow entrance, expanding basally (**Figure 4A**), and containing a small volume of nectar. The cells lining the cuniculus were flat or slightly convex (**Figures 4B–G**), thick-walled (5.97 μm ± 1.30), and had a thick (1.75 μm ± 0.47), intact cuticle. Secreted residues were visible on the cuticle using LM, SEM and TEM (**Figures 4C,E,G,H**). Both inner and outer tangential walls of the small epidermal cells, and those of 1-2 layers of the larger subepidermal cells, were thickened (**Figures 4B,E,F**) and lamellate (**Figures 4G,H**), the tissues closely resembling lamellar collenchyma. Cavities present in the middle lamellae of epidermal cells contained similar electron-dense material to that deposited on the surface of the cuticle (**Figures 4G,H**). Epidermal and subepidermal cells were similar in structure in that they both contained a centrally located vacuole and parietal cytoplasm, together with a large nucleus, and small plastids with osmiophilic, electron-dense globules (**Figures 4G,H**). Mitochondria and ER arrays were abundant in parietal cytoplasm, and secretory vesicles fused with the plasmalemma. The cells were interconnected by means of numerous primary pit-fields containing plasmodesmata (**Figure 4G**), and such connections were also present between epidermal, subepidermal, and ground parenchyma cells. Through the parenchyma ran three vascular bundles (**Figure 4D**). Starch was present in the ground parenchyma (**Figure 4F**), and chloroplasts were present in the hypodermis and ground parenchyma.

**FIGURE 5:**
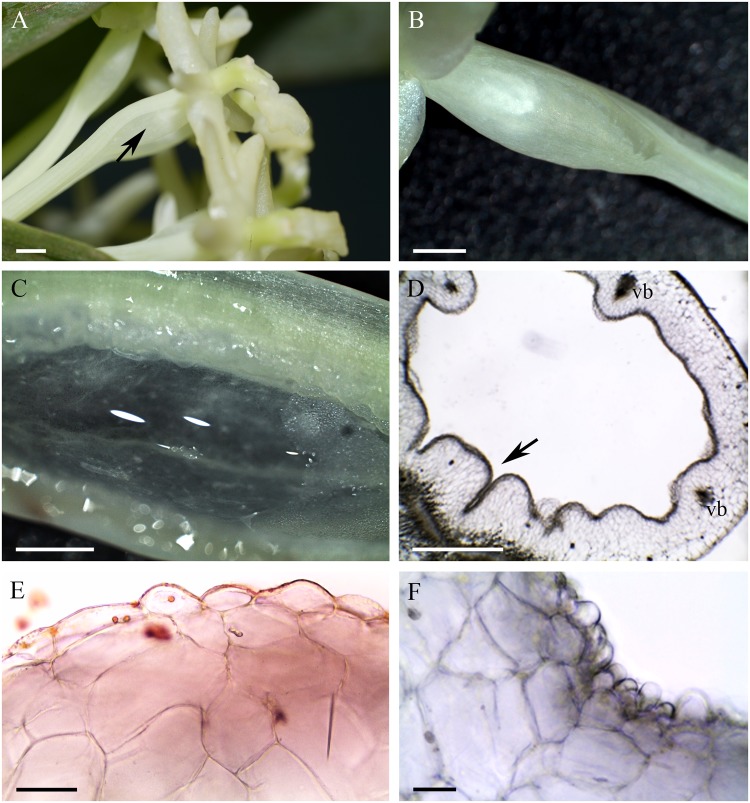
*Epidendrum vesicatum*. **(A)** Habit of the flower, cuniculus marked by arrow. **(B)** Lateral view of cuniculus. **(C)** Longitudinal section of cuniculus containing copious nectar. **(D)** Transverse section of cuniculus stained with IKI showing secretory tissue (arrow) adjacent to the transmitting tract. **(E)** Transverse section of secretory tissue stained with Sudan III. Note the thin cuticle present on secretory cells. **(F)** Detail of transverse section stained with IKI; note papillose epidermal cells and the absence of starch grains. Scale bars: A,B = 2mm, C,D = 1 mm; E,F = 20 μm.

The cuniculus of the greenish-white flowers of *Epidendrum vesicatum* measured ca. 10 mm in length (**Figures 5A,B**). The flowers produced a pleasant fragrance at night. The lumen of the cuniculus was oval in transverse section and tissues were translucent. The flower produced copious amounts of nectar which, owing to the transparency of the tissues, could easily be observed (**Figure 5C**). Secretory tissue was dorsally located in the cuniculus (**Figure 5D**). This region lay adjacent and parallel to the transmitting tract and ovary. The dorsal position of secretory tissue was observed only in *E. vesicatum*. The remaining area inside the cuniculus was non-secretory. Nectary tissue was composed of epidermal cells and subepidermal parenchyma. Epidermal cells enclosing the cuniculus in the nectary region were convex with large, centrally located vacuoles and parietal cytoplasm (**Figures 5E,F**). These cells had thin walls and a thin layer of smooth cuticle (**Figure 5E**), in contrast to the non-secretory area of the cuniculus, where cell walls were associated with a thicker layer of cuticle (not shown). Treatment with IKI revealed the absence of starch grains in nectary cells (**Figure 5F**). Three collateral vascular bundles supplied the ground parenchyma of the cuniculus (**Figure 5D**).

### Nectarless Species With no Nectar Visible on Macroscopic Observation

**FIGURE 6:**
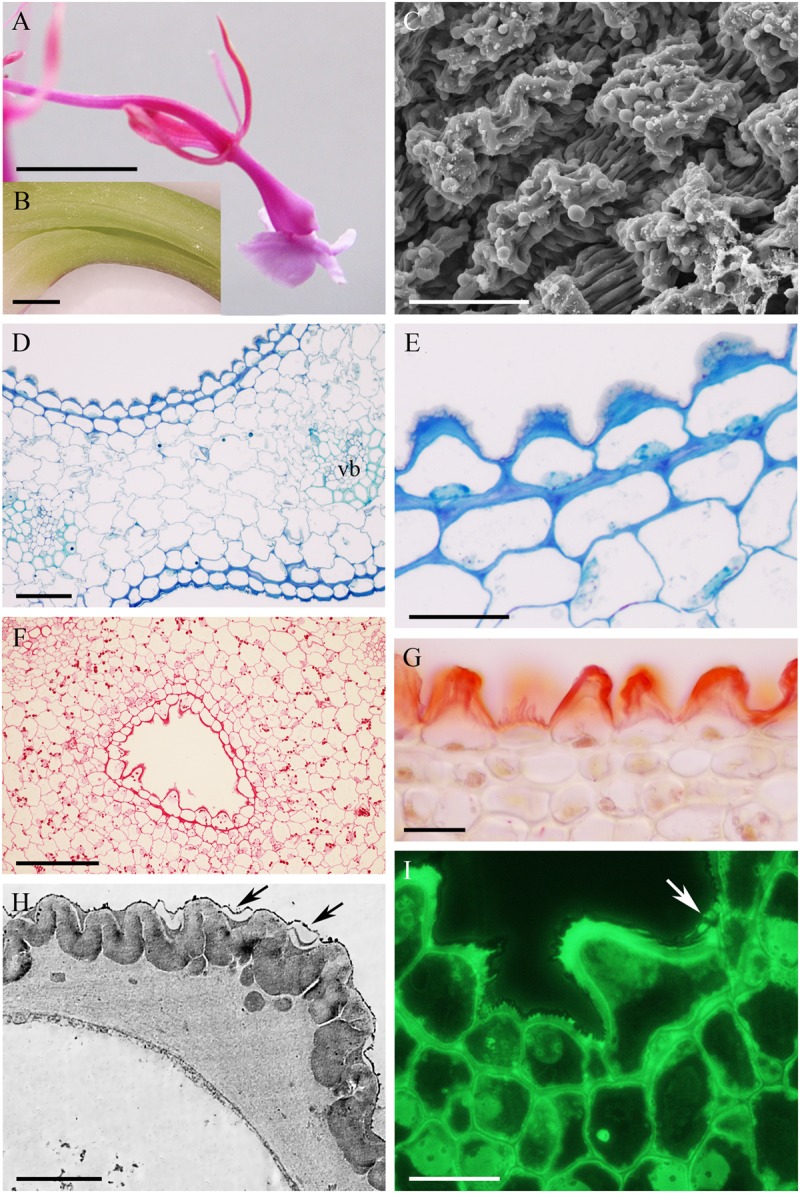
*Epidendrum capricornu*. **(A)** Habit of the flower. **(B)** Longitudinal section showing cuniculus. **(C)** Globular blisters on epidermal cells lining cuniculus. **(D)** Conical cells of epidermis enclosing cuniculus and parenchyma with vascular bundles (MB/AII). **(E)** Detail showing epidermis and subepidermal parenchyma. Note the thick, tangential walls of the epidermal cells and the convoluted cuticle. **(F)** PAS reaction stains copious starch present in papillose epidermis and subepidermal parenchyma. **(G)** Cuticle stained with Sudan III. **(H)** Outer epidermal cell wall with globular electron-dense material between cellulosic microfibrils, and blistered cuticle (arrows). **(I)** Section showing conical, epidermal cells and subepidermal parenchyma. Globules of secretion indicated by arrow. Scale bars: A = 1 cm; B = 1 mm; C,E,G,I = 20 μm; D = 50 μm; F = 200 μm; H = 2 μm.

The cuniculus of the non-fragrant, pink flowers of *Epidendrum capricornu* (**Figure 6A**) was wide at its entrance and tapered toward its base (**Figure 6B**), and measured 15 mm in length. The epidermal cells were conical close to the entrance, but papillose toward the base of the cuniculus, particularly on the side adjacent to the transmitting tract (**Figures 6C–G,I**). The striate cuticle of epidermal cells (0.90 μm ± 0.15 thick) lacked pores, but copious globular blisters were visible on its surface, when viewed by SEM and TEM (**Figures 6C,H**). Blisters with underlying material were also visible in sections stained with auramine O (**Figure 6I**). The tangential walls of both epidermal cells and the underlying parenchyma cells were cellulosic and thick (2.54 μm ± 1.02), but toward the tapered end of the cuniculus, cell walls were thinner. TEM observations indicated the presence of intravacuolar electron-dense, phenolic-like material (not shown). Similarly, electron-dense material was also observed to occur between the cellulosic microfibrils of the outer, tangential cell wall, and beneath the blistered cuticle (**Figure 6H**). Both epidermal and subepidermal parenchyma cells possessed a large central vacuole and a thin layer of parietal cytoplasm (**Figures 6E,I**), and accumulated starch (**Figure 6F**). The cuniculus was supplied with three collateral vascular bundles (**Figure 6D**).

**FIGURE 7:**
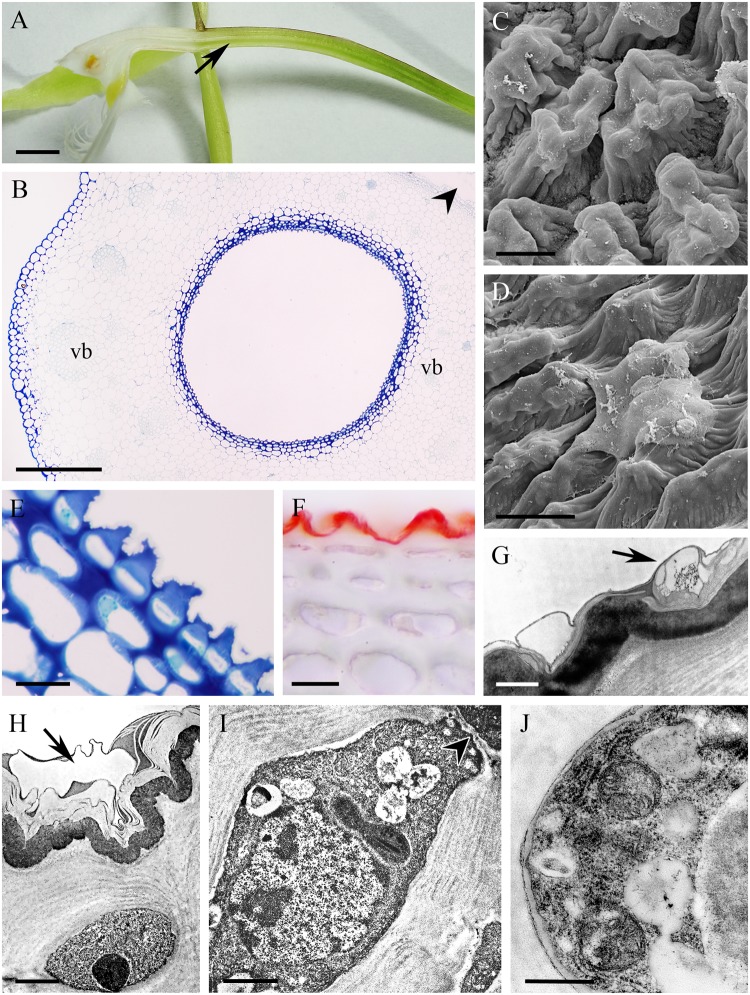
*Epidendrum ciliare.* **(A)** Longitudinal section of flower. Cuniculus indicated by arrow. **(B)** Transverse section of ovary with cuniculus and surrounding parenchyma containing vascular bundles (MB/AII). Transmitting tract is indicated by arrowhead. **(C)** Papillose epidermal cells lining cuniculus. **(D)** Secretion coating epidermis. **(E,F)** Epidermis and subepidermal parenchyma of cuniculus (MB/AII and Sudan III, respectively). Note thick tangential cell walls and cellulose projections penetrating cuticle. **(G)** Cell wall with thick, bilayered cuticle and cuticular blisters containing secretion (arrow). **(H)** Surface secretion and cuticular blister (arrow) of epidermal cell. **(I)** Electron-dense protoplast of epidermal cell containing large nucleus, starchless plastid and small vacuoles with flocculent content. Plasmodesmata in anticlinal cell wall marked with arrowhead. **(J)** Detail of parietal cytoplasm with profiles of ER, mitochondria and secretory vesicles. Scale bars: A = 1 cm; B = 500 μm; C = 10 μm; D–F = 20 μm; G = 1 μm; H, I = 2 μm; J = 0.5 μm.

The cuniculus of the white, fragrant flowers of *Epidendrum ciliare*, measured 45 mm in length (**Figure 7A**). Epidermal cells enclosing the cuniculus were flattened at its entrance and papillose toward its base (**Figures 7B–F**). The cuticle present on the epidermal papillae was ridged at their apices, but finely striate on the sides of the papillae (**Figures 7C,D**), and was 1.99 μm ± 0.52 thick. Despite the apparent absence of nectar during macroscopic investigations, surface secretion that resembled nectar and that coated the apical parts of the papillae was visible under SEM (**Figure 7D**). It was also observed by TEM to collect beneath the cuticular distensions (**Figures 7G,H**). The epidermal cells and the underlying 3-4 layers of parenchyma cells were smaller than those of the ground parenchyma cells through which ran several vascular bundles. In transverse section, epidermal cells and several layers of subepidermal cells were seen to possess thick (4.44 μm ± 0.99) tangential, cellulosic walls (**Figures 7E,F,H,I**), Such walls were particularly pronounced opposite the transmitting tract (**Figure 7B**). Numerous primary pit-fields with plasmodesmata in anticlinal and periclinal walls connected epidermal and subepidermal parenchyma cells (**Figures 7E,I**). TEM investigations showed the cuticle to be bilayered, having an outer lamellate layer and inner electron-dense and reticulate layer. Both these layers were highly convoluted (**Figure 7H**). The protoplasts of epidermal and subepidermal cells were electron-dense and contained numerous mitochondria, dictyosomes, ER profiles and secretory vesicles (**Figures 7I,J**). Small vacuoles containing vesicles or flocculent material were present (**Figure 7J**), and the larger vacuoles of the ground parenchyma had similar contents. The plastids contained an electron-dense stroma and few lamellae. Generally, these last organelles did not contain starch, but occasionally, starch grains were observed in parenchyma cells adjacent to vascular bundles. Chloroplasts were abundant in ground parenchyma cells. Numerous collateral vascular bundles of variable size were scattered throughout the ground parenchyma (**Figure 7B**). Lipids were detected exclusively in the cuticular layer (**Figure 7F**).

**FIGURE 8:**
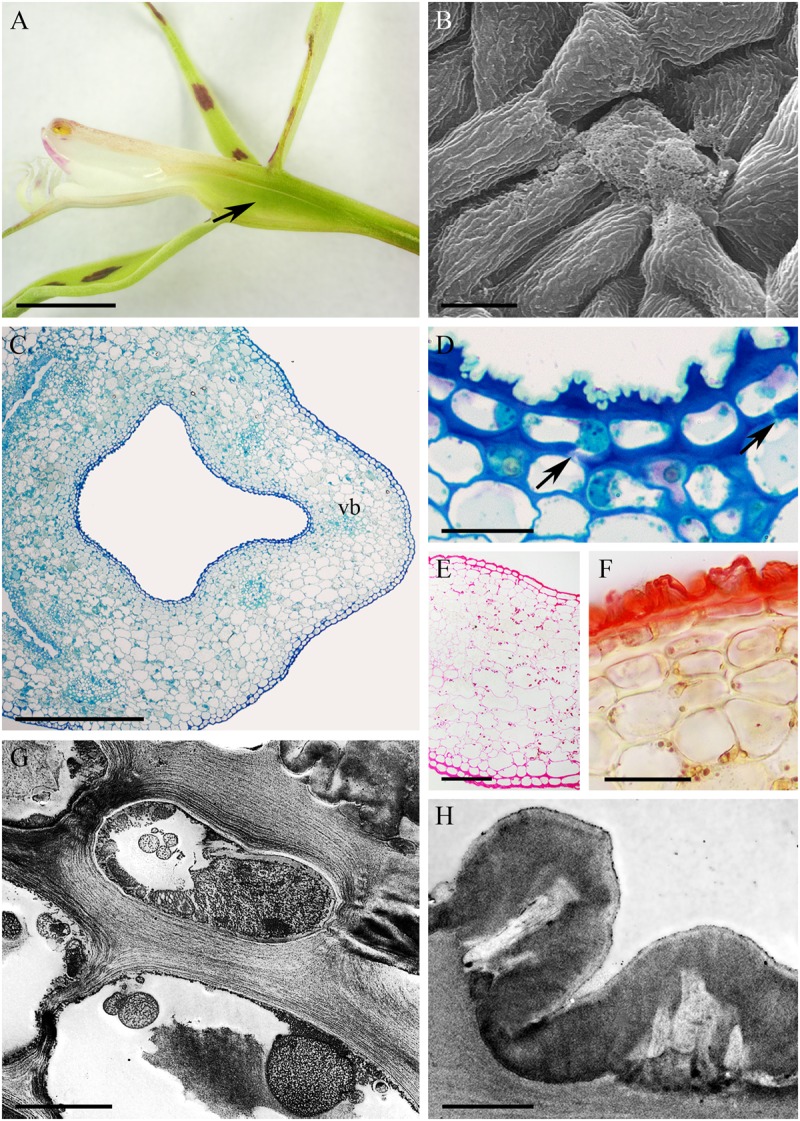
*Epidendrum criniferum*. **(A)** Longitudinal section of flower, the expanded part of the cuniculus indicated by arrow. **(B)** Surface secretion on cuticle. **(C)** Transverse section of ovary showing cuniculus and surrounding tissues (MB/AII). **(D)** Epidermis and subepidermal parenchyma with primary pit-fields (arrows). Cellulosic projections of cell wall with overlying cuticle (MB/AII). **(E)** PAS reaction shows starch in subepidermal parenchyma. **(F)** Thick cuticle stained with Sudan III. **(G)** Epidermal and subepidermal cells with intravacuolar, globular material. **(H)** Convoluted cuticle with cellulosic projections of cell wall. Scale bars: A = 4 mm; B = 50 μm; C = 500 μm; D = 20 μm; E = 100 μm; F = 30 μm; G = 5 μm; H = 2 μm.

Flowers of *Epidendrum criniferum* lacked fragrance, were greenish-white and spotted with magenta. The cuniculus measured 15.2 mm in length. It formed a wide reservoir below the entrance (**Figure 8A**) and tapered distally. The cuniculus was lined with flat or slightly convex epidermal cells that possessed a convoluted or ridged cuticle (**Figures 8B–H**) 1.24 μm ± 0.23 thick. Traces of secretory material were visible on the surface of the cuticle, when viewed by SEM (**Figure 8B**). The epidermal cells had thick tangential walls (4.34 μm ± 1.03), whereas those of the subepidermal and ground parenchyma were thin (**Figures 8D–G**). Epidermal and subepidermal cells contained a narrow layer of parietal cytoplasm and a large, central vacuole containing globular material (**Figure 8G**). Strands of cellulosic wall microfibrils occurred beneath the cuticular ridges (**Figure 8H**). Starch was present in both subepidermal and deeply located ground parenchyma cells (**Figure 8E**), whereas chloroplasts occurred only in the latter. The cuniculus was supplied with three collateral vascular bundles (**Figure 8C**).

**FIGURE 9:**
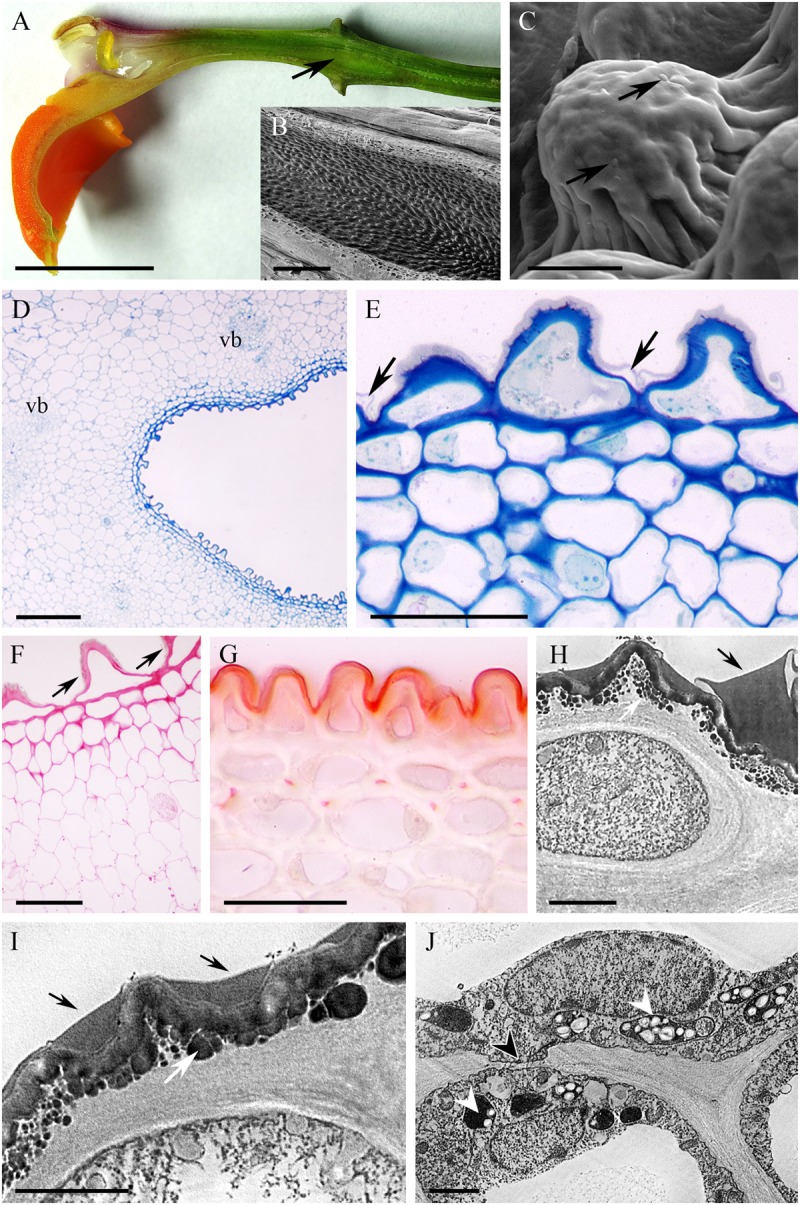
*Epidendrum pseudepidendrum*. **(A)** Longitudinal section of flower, cuniculus indicated by arrow. **(B)** Papillose epidermal cells lining cuniculus. **(C)** Detail of epidermal cell with cuticular blisters (arrows). **(D)** Epidermis and parenchyma with vascular bundles enclosing cuniculus (MB/AII). **(E)** Detail of epidermis and subepidermal parenchyma of cuniculus. Note secreted material beneath cuticle (arrows). **(F)** PAS reaction stains cell walls and starch in ground parenchyma cells. Surface material accumulates between cuticular ridges (arrows). **(G)** Sudan III stains cuticle and intercellular material. **(H)** Epidermal cell with surface secretion (arrow). **(I)** Detail of epidermal cell wall with subcuticular globular material (white arrow) and surface secretion (black arrows). Secretory vesicles fusing with plasmalemma are also visible. **(J)** Parenchyma cell with plastids containing an electron-dense stroma and starch grains (arrowheads). A primary pit-field with plasmodesmata marked with arrowhead. Scale bars: A = 1 cm; B,D = 200 μm; C = 10 μm; E,G = 40 μm; F = 50 μm; H,J = 2 μm.

The cuniculus of the orange and green, non-fragrant flowers of *Epidendrum pseudepidendrum* was 38 mm long. It had a very narrow entrance expanding to form a wider region at the level of insertion of the perianth segments (**Figure 9A**). The epidermis at the mouth of the cuniculus was papillose, the papillae being longer toward its base (**Figures 9B–G**). The cuticle overlying the papillae was thick (0.45 μm ± 0.06) and formed blisters and distensions (**Figures 9C,E**). Surface secretory material was present apically and between cuticular ridges (**Figures 9E,F,H,I**). This material, which stained with Sudan III, was also present in intercellular spaces (**Figure 9G**). The walls of epidermal cells and 1-2 layers of the subepidermal tissue were 1.57 μm ± 0.30 thick and cellulosic (**Figures 9D–I**). Three large and several small collateral vascular bundles supplied the ground parenchyma (**Figure 9D**). Observations using TEM revealed that epidermal cells contained a large nucleus and electron-dense, granular cytoplasm with mitochondria and secretory vesicles, the last fusing with the plasmalemma (**Figure 9I**). Plastids with starch and/or an electron-dense stroma were present in subepidermal and ground parenchyma cells (**Figures 9F,J**), whereas chloroplasts occurred only in ground parenchyma cells. Lipid bodies were occasionally observed in epidermal cells. Primary pit-fields with plasmodesmata (**Figure 9J**) were present in periclinal walls between epidermal and subepidermal cells.

**FIGURE 10:**
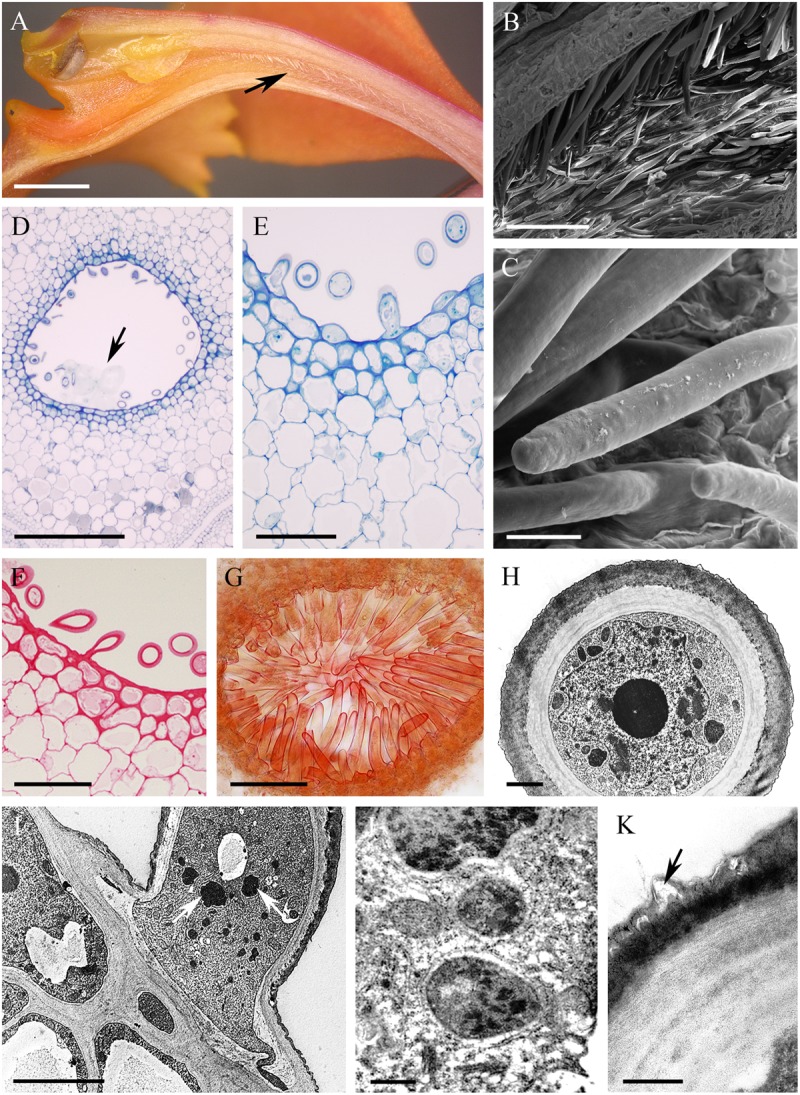
*Epidendrum radicans*. **(A)** Longitudinal section of flower, the cuniculus indicated by an arrow. **(B)** Unicellular trichomes lining cuniculus. **(C)** Detail of smooth cuticle of trichomes with small blisters. **(D)** Tissues enclosing cuniculus. Secreted surface material is marked by arrow (MB/AII). **(E)** Detail of epidermis of cuniculus with trichomes and subepidermal tissues. **(F)** PAS reaction stains cellulosic cell walls. **(G)** Sudan III stains thick cuticle of trichomes. **(H)** Transverse section of trichome showing cell wall with thick, smooth, but slightly blistered cuticle and electron-dense protoplast enclosing large nucleus. **(I)** Longitudinal section through trichome, epidermal and subepidermal cells. Note electron-dense plastids (arrows) in trichome. **(J)** Detail of cytoplasm of trichome with starchless plastids, mitochondria, dictyosomes and ER. **(K)** Cell wall of trichome with blistered cuticle (arrow). Scale bars: A = 2 mm; B = 200 μm; C = 20 μm; D = 500 μm; E,F = 50 μm; G = 100 μm; H = 2 μm; I = 5 μm; J,K = 0.5 μm.

The cuniculus of the non-fragrant, orange flowers of *Epidendrum radicans* measured 25 mm in length. The epidermal cells at its entrance were papillose. Of the investigated taxa, this species was unique in that the cuniculus was lined with unicellular trichomes of average length 132 μm. These epidermal trichomes arose from just below the entrance to the cuniculus and were distributed along its length to the base (**Figures 10A–I**). They had a smooth and thick (1.55 μm ± 0.89) cuticle (**Figures 10C,H**). Observations of the cuticle by means of SEM, LM and TEM revealed the presence of surface material, as well as cuticular distensions (**Figures 10C,K**). Cell walls of trichomes were 1.21 μm ± 0.25 thick. Epidermal and subepidermal cells were small compared with those of the underlying ground parenchyma, the cell walls being only slightly thickened (**Figures 10D–F,I**). These walls were 0.91 μm ± 0.19 thick and had a thin cuticle (0.26 μm ± 0.07). The epidermal cells, including the unicellular trichomes, had dense protoplasts containing a large nucleus and small vacuoles (**Figures 10E,H–J**). Mitochondria, ER profiles, dictyosomes and secretory vesicles were predominant in the cytoplasm of trichomes and subepidermal parenchyma cells. The plastids contained an electron-dense stroma, densely packed stacks of lamellae and plastoglobuli, but no starch. Starch, however, was present in the plastids of ground parenchyma. Chlorophyll was not detected by FM in parenchyma cells surrounding the cuniculus. The ground parenchyma was supplied by three collateral vascular bundles (not shown).

**FIGURE 11:**
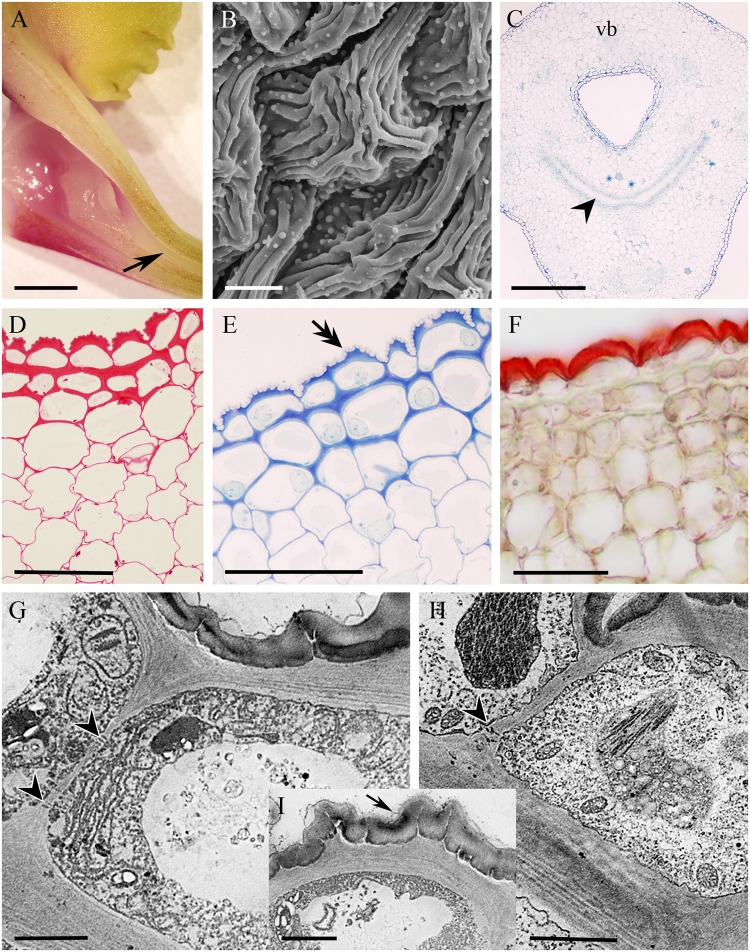
*Epidendrum xanthoianthinum*. **(A)** Longitudinal section of flower, the cuniculus indicated by arrow. **(B)** Ridged surface of blistered cuticle of epidermal cells. **(C)** Transverse section of ovary showing cuniculus and associated tissues, the transmitting tract indicated by arrowhead (MB/AII). **(D)** PAS reaction stains cell walls and sparse starch grains. **(E)** Detail of epidermis and subepidermal parenchyma. Note that cellulosic projections of the cell wall (double arrow) penetrate the cuticle (MB/AII). **(F)** The thick cuticle stains selectively with Sudan III. **(G)** Parietal cytoplasm of epidermal cell, showing profiles of ER and plastids. Vesicles are present in the vacuole. Surface secretion is visible on the cuticle. **(H)** Plastids with dense stroma and minute starch grains in epidermal cells. In **G,H**, plasmodesmata in anticlinal walls are marked with arrowheads. **(I)** Detail of cell wall penetrating cuticle and secreted surface material (arrow). Scale bars: A = 2 mm; B = 10 μm; C = 300 μm; D–F = 50 μm; G–I = 2 μm. vb, vascular bundle.

The cuniculus of the non-fragrant, yellow, rose and green flowers of *Epidendrum xanthoianthinum* measured 14 mm long. Its entrance was wide, but the lumen tapered toward the base (**Figure 11A**). The epidermis enclosing the cuniculus was composed of slightly convex cells possessing a convoluted or ridged cuticle (**Figures 11B–G,I**) 1.87 μm ± 0.27 thick. Abundant cuticular blisters were visible under SEM (**Figure 11B**), and in TEM, these appeared electron-translucent (**Figures 11G,I**). The tangential cellulosic walls of the epidermal cells and one layer of the subepidermal parenchyma were slightly thickened (**Figures 11D–H**), those of the epidermis being 2.28 μm (±0.62) thick. The epidermal cells contained intensely staining cytoplasm, together with a large nucleus and plastids containing small starch grains (**Figures 11D,E,G**). Moreover, TEM investigations revealed the presence of numerous lamellae and plastoglobuli (**Figure 11H**) within these plastids. The cytoplasm also contained arrays of ER, as well as dictyosomes and secretory vesicles (**Figures 11G,H**). The tissues surrounding the cuniculus were supplied with three large, and several small collateral vascular bundles (**Figure 11C**).

## Discussion

Species of *Epidendrum* investigated in this study varied greatly in terms of flower size, color, fragrance, and also in the size and shape of the cuniculus. All these characters may reflect the type of pollinator associated with each species. Whereas some of the flowers are obviously nectariferous and produce copious nectar (*E. difforme*, *E. nocturnum* and *E. vesicatum*), others produce smaller volumes of nectar (*E. porpax* and *E. rigidum*), whereas yet others seemingly produce none (*E. capricornu*, *E. ciliare*, *E. criniferum*, *E. pseudepidendrum*, *E. radicans*, *E. xanthoianthinum*). Although evidence for nectar secretion has previously been recorded for Laeliinae, the amount produced is frequently small, making it difficult to measure volume and sugar concentration (Krahl et al., 2017). Conversely, *E. vesicatum* and *E. nocturnum* produce copious amounts of nectar, which can be easily collected with micro-syringes, and its sugar concentration measured with a refractometer. *Epidendrum vesicatum*, whose flowers are adapted to pollination by nocturnal moths, produces 9–15 μL of dilute nectar of concentration 5–7% sugar (E.R. Pansarin, unpublished data).

In the absence of a nectar reward, approximately one-third of investigated orchid species rely on various kinds of deception or mimicry to attract pollinators (Cozzolino and Widmer, 2005; Jersákowá and Johnson, 2006; Gaskett, 2011), and many reports have indicated that deception was the ancestral condition in Orchidaceae (Hobbhahn et al., 2013; Johnson and Schiestl, 2017, and references therein). According to Cozzolino and Widmer (2005) and Jersákowá et al. (2006), deception can, under certain circumstances, be advantageous in that it enables conservation of resources and discourages repeated visits by pollinators, thereby promoting outcrossing. Food-deception has been reported for Laeliinae, including *Epidendrum* (Boyden, 1980; Almeida and Figueiredo, 2003; Pansarin and Amaral, 2008b; Fuhro et al., 2010; Vega and Marques, 2015). Indeed, according to Ackerman (1986), *Epidendrum* is primarily a food deceptive genus. This is supported by the work of Hágsater and Soto-Arenas (2005) which reports that many members of *Epidendrum*, e.g., the *Epidendrum secundum* complex, do not offer any nectar reward, with some observations indicating that many *Epidendrum* spp. display attributes of food-deceptive taxa, such as infrequent visits by pollinators and low fruit set (Ackerman and Montalvo, 1990; Almeida and Figueiredo, 2003; Pansarin and Amaral, 2008b; Fuhro et al., 2010; Pinheiro et al., 2010, 2011). Therefore, in future, it will be necessary to investigate nectar production by certain taxa, including members of the *Epidendrum secundum* complex, more thoroughly and critically, since it is now known that the flowers of some species of *Epidendrum*, once thought to employ nectar deception pollination strategies (e.g., *E. puniceoluteum*), in fact, have nectar-secreting epidermal papillae, and that nectar is collected from them by both hummingbirds and butterflies (E.R. Pansarin, pers. obs.). Remarkably, some species of *Epidendrum* that lack floral nectar possess extra-floral nectaries. The production of extra-floral nectar is generally considered a defense strategy in that it encourages ants to patrol plants, thus discouraging herbivory (Delabie, 1995; Almeida and Figueiredo, 2003). Even so, since *E. nocturnum* produces copious nectar, yet was the only species investigated in this study to have extra-floral nectaries, their presence is clearly not restricted exclusively to non-rewarding species.

The rewardless status and deceptive pollination systems proposed for a number of species on the basis of macroscopic observations alone is entirely understandable since, in fact, no nectar whatsoever was visible using this technique for *E. capricornu*, *E. criniferum*, *E. pseudepidendrum*, *E. radicans*, and *E. xanthoianthinum*. Nevertheless, close observations of cells lining the cuniculus in presumed rewardless species by LM, SEM and TEM revealed the presence of nectar-like residues and established that they possess an organelle complement typical of secretory cells. As well as similarities in the ultrastructure of these cells and the presence of thick tangential cell walls (with the exception of *E. vesicatum*), other secretory characters particularly pronounced in *E. nocturnum, E. ciliare, E. radicans* and *E. xanthoianthinum* included the abundant mitochondria, ER and secretory vesicles. Furthermore, cell wall protuberances were present in *E. nocturnum,* indicating intense transmembrane transport. The cuticle overlying the epidermal cells lining the cuniculus in these species was blistered, and secretory material had accumulated beneath and upon its surface. No relationship was found to occur between the thickness and structure of the cuticle in both species with copious nectar occurring in the cuniculus, and those exhibiting only residues of secreted surface material. Of the investigated species, only the cuniculus of *E. radicans* was lined with unicellular trichomes, and in this respect, it resembled that of *E. fulgens*, whose anatomy was studied by Moreira et al. (2008). These authors proposed, owing to the dense cytoplasmic content of the trichomes, that the latter are highly metabolically active and thus, probably involved in secretion, even though no nectar was found within the cuniculus. Similarly, in *E. radicans*, the organelle complement of such trichomes, coupled with the presence of surface material, indicated that they too are secretory.

We did not find any floral food-rewards other than nectar in species investigated in this study. In our opinion, the presence of nectar-like surface residues during detailed microscopical investigations is indicative of, at the very least, a limited degree of nectary activity, and it may be that meager volumes of nectar are sufficient to maintain the interest of pollinators. According to Ackerman and Montalvo (1990), *E. ciliare* is self-compatible, but outcrossed and pollinated by the moth *Pseudosfinx tetrio*. During experimentally augmented pollination, fruit-set increased in the short-term, but in subsequent seasons, it declined significantly, since greater fruit production demanded greater resources. Consequently, a large number of pollination events and investment in the production of large volumes of nectar do not always benefit the plant. Another explanation for the absence of nectar from the cuniculus of species predominantly visited by nocturnal pollinators is that nectar secretion occurs only at night and is reabsorbed during the day. In fact, based on floral characters and the release of fragrance at night, many species of *Epidendrum* are believed to be pollinated by nocturnal moths (van der Pijl and Dodson, 1969; Pansarin and Pansarin, 2010), and this has been confirmed by investigations of their reproductive biology (Pansarin, 2003; Pansarin and Pansarin, 2017).

Relatively numerous globular blisters were observed by SEM on the cuticle of epidermal cells lining the cuniculus of *E. capricornu* and *E. xanthoianthinum*. TEM observations indicated that they are delimited delimited by a thin layer of cuticle bearing almost electron-translucent material that probably represents nectar. It is thus likely that the abundant cuticular blisters present in these two species were probably the result of epidermal secretory activity.

*Epidendrum pseudepidendrum* is regarded to be a humming-bird-pollinated and rewardless species (van der Pijl and Dodson, 1969; van der Cingel, 2001). Nevertheless, as far as we are aware, there are no experimental data to support this assertion. If, however, this is true, it would pose an interesting conundrum, since bird-pollinated flowers usually offer nectar. Although we demonstrated the presence of surface secretion in this species, owing to its osmiophilic nature, this secretion evidently is not a simple sugar solution. Nectar, far from being merely a dilute aqueous solution of sugars, may also contain other compounds such as amino acids, lipids and secondary metabolites, some of which are osmiophilic. Therefore, we cannot rule out the possibility that *E. pseudepidendrum* is indeed nectariferous.

In most of the obviously nectariferous taxa investigated, the epidermis of the cuniculus was relatively glabrous, whereas in seemingly nectarless species, it was predominantly papillose or trichomatous. This is not congruent with the nectary studies undertaken for some members of Orchidoideae, where nectar secretion was shown to be positively correlated with the presence of papillae (Bell et al., 2009). Trichomes and papillae were also present in the nectaries of other genera of Epidendroideae, such as *Oeceoclades* (Aguiar et al., 2012), *Ascocentrum* (Stpiczyńska et al., 2011), and representatives of Laeliinae, such as *Encyclia* (Krahl et al., 2017) and *Brassavola flagellaris* (Stpiczyńska et al., 2010). The presence of epidermal papillae and trichomes has been considered a strategy for increasing the surface area for nectar secretion/reabsorption (Stpiczyńska, 2003; Stpiczyńska et al., 2005). Since it is likely that all species of *Epidendrum* investigated in this study secrete nectar to a greater or lesser degree, it is not possible to correlate nectar production with the presence of papillae/trichomes. Nevertheless, the possibility that the increased surface area of the epidermis lining the cuniculus may account for the seemingly nectarless status of certain species, cannot be discounted, since the secreted nectar may be reabsorbed more efficiently. It is worth stating, however, that the presence of papillae is not necessarily exclusively related to nectar secretion/reabsorption. For example, papillae present in the spur of deceptive orchids such as *Dactylorhiza* (Bell et al., 2009) probably provide tactile cues for insect visitors.

In all investigated species (with the exception of *E. vesicatum*), epidermal and subepidermal parenchyma cells had thick, tangential cellulosic cell walls, and in the case of the outer epidermal walls, cellulosic projections extended as far as, and traversed the thick cuticle, possibly facilitating the transport of secretion across the latter. Such thick cellulosic walls are characteristic of collenchyma. Collenchymatous cell walls have also been recorded for the nectaries of other species of *Epidendrum* (e.g., Pansarin and Amaral, 2008b; Vieira et al., 2017), the cuniculus of *Brassavola flagellaris* (Stpiczyńska et al., 2010), the nectaries of putatively ornithophilous *Maxillaria coccinea* (Stpiczyńska et al., 2004) and *Ornithidium* *sophronitis* (Stpiczyńska et al., 2009), and some Aeridiinae (Stpiczyńska et al., 2011). Here, collenchyma may prevent mechanical damage to the nectary tissues, and also facilitate apoplastic transport of nectar. Numerous pit-fields with plasmodesmata traversing anticlinal cell walls between epidermal cells, as well as periclinal walls between epidermal and parenchyma cells, may also be indicative of symplastic transport. Abundant plasmodesmata connections have also been reported for the nectary tissues of other plant species having thick collenchymatous cell walls, such as *M. coccinea* (Stpiczyńska et al., 2004), as well as those with thin-walled nectary cells (e.g., Nepi, 2007; Stpiczyńska et al., 2011, 2018). Our results generally agree with the model proposed by Vassilyev (2010) for the functioning of the nectary. According to this author, nectar moves by a pressure-driven mass flow along an apoplastic route, but pre-nectar sugars are transported from the phloem via the symplast to the secretory cells, where finally, nectar is formed, and sugars are actively transported across the plasmalemma by eccrine secretion. Since abundant secretory vesicles were present in secretory cells of the investigated species, both close to the plasmalemma and in the parietal cytoplasm, we propose that granulocrine secretion also operates in nectary cells of *Epidendrum*.

The thick cell walls of *E. nocturnum* were particularly remarkable in their possession of wall protuberances thought to improve efficient transport across the plasmalemma. Although cell wall protuberances have frequently been reported for the nectaries of other angiosperms (Fahn, 1979; Kronestedt-Robards and Robards, 1991), they have rarely been recorded for Orchidaceae (Pais and Figueiredo, 1994; Stpiczyńska et al., 2018). The involvement in intense secretory activity of the epidermal cells enclosing the cuniculus of investigated species of *Epidendrum* is confirmed by the presence of numerous secretory vesicles that gather next to the plasmalemma, the extensive arrays of endoplasmic reticulum, the abundant mitochondria, and the dictyosomes, as well as invaginations of the plasmalemma that increase the surface area for secretion and possible reabsorption of nectar. In future, we intend performing ultrastructural studies on the thin-walled nectary cells of *E. vesicatum*, in order to assess how well they are structurally adapted for nectar secretion.

We did not measure the volume of secreted nectar in the present project, but based on microscopical observations, we found no connection between the number and distribution pattern of vascular bundles present in the parenchyma and secretory activity. However, the secretory status and nectary activity of all species investigated are further supported by the distribution of abundant starch predominantly located near the main vascular bundles supplying the nectary, but also, in some cases, within epidermal and subepidermal parenchyma cells. The importance of starch has been widely reported for the floral secretory tissues (in particular, nectaries) of many taxa, including orchids, and it has been proposed that hydrolysis of starch reserves provides both the metabolic energy for the secretory process and the sugars for nectar production (Pacini and Nepi, 2007). In the majority of investigated species, parenchyma cells containing chloroplasts were also able to synthesize sugars, whereas plastids within the secretory epidermis frequently possessed an electron-dense stroma, indicating that they might be engaged in the synthesis of various secondary metabolites, including alkaloids (Facchini, 2001), that are frequently present in nectar. Many species of *Epidendrum* are visited by butterflies. Some male lepidopterans are attracted by pyrrolizidine alkaloids that are used in mating (van der Cingel, 2001 and references therein; Hágsater and Soto-Arenas, 2005 and references therein) and in the synthesis of their pheromones. In many plant species, alkaloids are a common constituent of nectar (Masters, 1991; Adler and Irwin, 2005; Nepi, 2007; Manson et al., 2013), and since *E. difforme* is visited by male Ctenuchidae and Noctuidae moths (Goss, 1977), it is possible that its nectar also contains alkaloids. The occurrence of pyrrolizidine alkaloids has been considered a factor involved in attracting Ithomiinae butterflies to the flowers of *E. paniculatum* (van der Pijl and Dodson, 1969). According to Pansarin (2003), a species closely related to *E. paniculatum* (namely, *E. densiflorum*), is also pollinated by Ithomiinae butterflies and Arctiidae moths, and both types of insect have been reported to collect alkaloids from flowers. However, tests for alkaloids showed these compounds to be absent from flowers of *E. densiflorum* (Pansarin, 2003). Detailed chemical analysis of *Epidendrum* nectar is now necessary.

## Conclusion

To conclude, we agree with Pinheiro and Cozzolino (2013) that the genus *Epidendrum* is an ideal model system for the comparative study of the association between pollination efficiency and the evolution of floral traits in both rewarding and deceptive orchids, especially since members of this enormous genus display diverse reproductive systems ranging from self-incompatibility to autogamy (Hágsater and Soto-Arenas, 2005). For example, autogamy has been reported for nectariferous *E. nocturnum* (Catling, 1990) and for *E. rigidum* (Iannotti et al., 1987). By contrast, self-incompatibility occurs in nectariferous *E. difforme* (Goss, 1977), whereas in self-compatible taxa, geitonogamy is thought to be restricted to species that do not offer any food-rewards to pollinators (Almeida and Figueiredo, 2003; Pansarin and Amaral, 2008b; Fuhro et al., 2010). Nevertheless, as our study indicates, perhaps the time has come to look more closely at whether *Epidendrum* spp. considered to lack food-rewards merely on the basis of macroscopic examination really are rewardless, and to investigate their floral biology in association with molecular studies, in order to explore the evolution and the production of floral food-rewards in this genus (Pansarin et al., 2012; Pansarin and Maciel, 2017). Furthermore, this should not be restricted to *Epidendrum*, but extended to other orchid genera also.

## Author Contributions

MS conceived the study, did the microscopy, prepared the draft version of manuscript, and contributed to the final version. MK contributed to the microscopy and documentation, and the final figures. KD developed, expanded, and contributed to the final version of manuscript. EP contributed to the microscopy and contributed to the final version of manuscript.

## Conflict of Interest Statement

The authors declare that the research was conducted in the absence of any commercial or financial relationships that could be construed as a potential conflict of interest.
